# Sub-Nucleocapsid Nanoparticles: A Nasal Vaccine against Respiratory Syncytial Virus

**DOI:** 10.1371/journal.pone.0001766

**Published:** 2008-03-12

**Authors:** Xavier Roux, Catherine Dubuquoy, Guillaume Durand, Thi-Lan Tran-Tolla, Nathalie Castagné, Julie Bernard, Agnès Petit-Camurdan, Jean-François Eléouët, Sabine Riffault

**Affiliations:** Unité de Virologie et Immunologie Moléculaires (UR892), INRA, Jouy-en-Josas, France; AIDS Research Center, Chinese Academy of Medical Sciences and Peking Union Medical College, China

## Abstract

**Background:**

Bronchiolitis caused by the respiratory syncytial virus (RSV) in infants less than two years old is a growing public health concern worldwide, and there is currently no safe and effective vaccine. A major component of RSV nucleocapsid, the nucleoprotein (N), has been so far poorly explored as a potential vaccine antigen, even though it is a target of protective anti-viral T cell responses and is remarkably conserved between human RSV A and B serotypes. We recently reported a method to produce recombinant N assembling in homogenous rings composed of 10–11 N subunits enclosing a bacterial RNA. These nanoparticles were named sub-nucleocapsid ring structure (N SRS).

**Methodology and Principal Findings:**

The vaccine potential of N SRS was evaluated in a well-characterized and widely acknowledged mouse model of RSV infection. BALB/c adult mice were immunized intranasally with N SRS adjuvanted with the detoxified *E. coli* enterotoxin LT(R192G). Upon RSV challenge, vaccinated mice were largely protected against virus replication in the lungs, with a mild inflammatory lymphocytic and neutrophilic reaction in their airways. Mucosal immunization with N SRS elicited strong local and systemic immunity characterized by high titers of IgG1, IgG2a and IgA anti-N antibodies, antigen-specific CD8^+^ T cells and IFN-γ-producing CD4^+^ T cells.

**Conclusions/Significance:**

This is the first report of using nanoparticles formed by the recombinant nucleocapsid protein as an efficient and safe intra-nasal vaccine against RSV.

## Introduction

Respiratory syncytial virus (RSV) is a member of the *Pneumovirus* genus in the *Paramyxoviridae* family that causes severe respiratory tract infections in infants less than two years old and in elderly or immuno-compromised patients. In fact, RSV induced-bronchiolitis is the most common cause of infant hospitalization in the developed world and is a suspected risk factor of recurrent wheeze and asthma in later life [1]. Despite the substantial health and economic burden caused by RSV illness, there is currently no vaccine available. Several factors are responsible for the lack of an effective and safe vaccination strategy.

Firstly, the viral infection itself does not lead to protective immunity and re-infections occur throughout life during winter epidemics. Besides, as there are various RSV isolates circulating throughout the world, from serotype A or B, an effective vaccine should protect against all of them. Secondly, RSV disease is largely immune mediated and any potential vaccine must avoid enhancing immunopathology in the lower respiratory tract. Indeed, in the sixties, a formalin-inactivated (FI)-RSV vaccine caused a dramatic increase in the severity of naturally acquired disease [2], [3]. Two children developed fatal vaccine-enhanced RSV disease, characterized by pulmonary neutrophilia and eosinophilia. Therefore an effective vaccine needs to limit viral replication without causing disease exacerbation due to the obstruction of the airways with inflammatory cells. Thirdly, the primary targets of a potential vaccine are infants 0–6 months old and issues such as the immaturity of their immune system, the presence of maternally-derived RSV neutralizing Abs and specific safety concerns must be addressed.

Since formalin-inactivated virus vaccines caused aggravated disease upon natural infection, a large array of alternative vaccination strategies have been tested against RSV during the last 40 years in terms of viral antigen, delivery system (live attenuated virus, replicating or non-replicating vectored vaccines, subunit vaccines), adjuvant and route of administration (parenteral or mucosal) [4]. These vaccination strategies were mostly evaluated in rodent experimental models of RSV infection and sometimes in non human primates or cattle (natural host of bovine RSV). Murine studies have been very useful for defining and characterizing immunogenic RSV antigens, and for deciphering the immune correlates of protection versus disease exacerbation [5].

Among the most immunogenic RSV proteins, the F and G envelope glycoproteins are the targets of neutralizing antibodies (Ab) [6], [7]. A variety of subunit, vectorized or DNA vaccines, targeting the F and G surface proteins of RSV have reached various stages of development [4]. However, recombinant G and F or chimeric FG were often found to cause enhancement of lung pathology upon RSV challenge, in association with the priming of Th2 cells [4]. Apart from the protective role of neutralizing antibodies, experimental studies in calves [8] and in mice [9] indicated that CD8^+^ T cells are required for the resolution of an acute primary RSV infection and are also protective against vaccine-driven lung eosinophilia following RSV infection [10], [11]. Internal viral proteins are the main antigenic targets of RSV-specific CTL responses, among which the nucleoprotein N is a major carrier of CTL epitopes in human and cattle [12]–[15]. Furthermore N is the most conserved viral protein between RSV human isolates and it even shares ≈94% amino acid identity between bovine and human RSV [16]. Thus a vaccine with N as an antigen might protect against both bovine and human RSV.

Contrary to F or G, the N nucleocapsid protein of RSV has been poorly explored as a vaccine antigen and, when so, only under the form of virus-vectorized [17], [18] or free DNA vaccine co-administered with plasmid DNA encoding the fusion protein F [19], [20]. These vaccination strategies efficiently triggered protective T cell responses. Nevertheless the protection afforded by recombinant vaccinia virus (rVV) expressing N against viral replication was not as strong as the one afforded by rVV-F or rVV-G [17]. Plasmid DNA vaccine encoding N and F conferred partial protection against RSV challenge in terms of clinical score and virus load in infant rhesus monkeys [19] or in calves [20], but the respective role of F and N viral immunogens in the protection was not investigated. One hypothesis for the moderate efficacy of N as a vaccine antigen may be its mode of delivery via plasmid DNA or rVV. Recently, a recombinant form of the nucleocapsid protein from the measles virus, another paramyxoviridae, was shown to prime protective anti-viral CD8^+^ CTL after a single topical immunization via the buccal mucosa [21]. Due to its strong tendency to form nucleocapsid-like super-structures, the N protein from RSV used to be difficult to produce in large amounts as a soluble recombinant protein. Recently, Tran et al. in our laboratory, have developed an original technology to produce and purify N, co-expressed in *E. coli* with the C-terminal fragment of the RSV phosphoprotein (PCT) fused to glutathione S-transferase. The strong interaction between PCT and N allows the purification of recombinant N, which assembles in homogenous rings (diameter 15 nm), composed of 10–11 N subunits entrapping a stretch of 70 nucleotide-long bacterial RNA. These nanoparticles were named “N SRS” for sub-nucleocapsid ring structures [22]. Thus we made the hypothesis that a N SRS sub-unit vaccine could provide better protection than the previous vaccination assays with N and safer protection than the previous F and G sub-unit vaccines.

Intranasal vaccination against RSV represents an attractive approach in order to prevent virus spreading to the lower respiratory tract since host immune cells are likely to be primed in the relevant regional lymphoid tissues. However, the mere introduction of non-replicating antigens into the respiratory tract has often proven insufficient at inducing T cell responses. Yet, co-administration of antigens with bacterial enterotoxins, like *Escherichia coli* heat labile enterotoxin (LT), promotes local and systemic Ab responses and augments Ag-specific T cell responses [23]. For instance, a mutant derivative of LT, named LT(R192G), when administered intra-nasally with viral epitopes/antigens was shown to induce mixed Th1 and Th2 cellular immunity, and CD8^+^ CTL responses [24]–[27].

In the present study, we present experimental data showing that N SRS nanoparticles can be rapidly engulfed by murine macrophage and dendritic cell lines. Furthermore we demonstrate that intranasal delivery of N SRS, together with the mucosal LT(R192G) adjuvant, primed adult BALB/c mice for Ab, CD4 and CD8 mediated T cell responses and conferred protective immunity against viral replication. Following virus infection, a mild inflammatory infiltration of the lungs by lymphocytes and neutrophils occurred, with activated CD4^+^ and CD8^+^ T cell subsets being both recruited to the airways.

## Results

### Fluorescent N SRS complexes are rapidly internalized by murine cell lines with antigen-presentation ability

**Figure 1:**
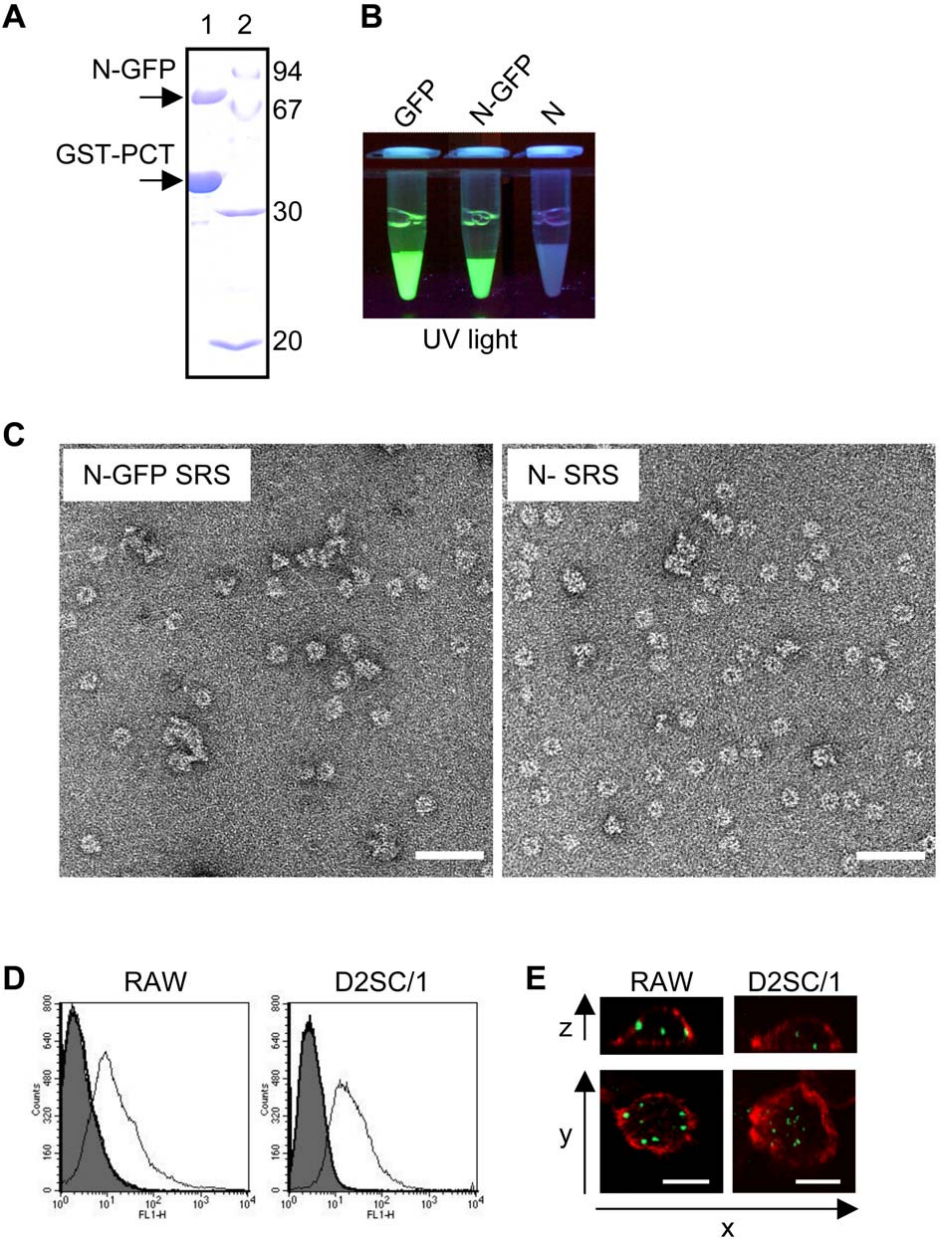
N-GFP fusion protein assembles into fluorescent SRS that are internalized by murine macrophage and dendritic cell lines. (A) SDS-PAGE analysis of purified GST-PCT+N-GFP complex. Sample (1) was denatured in Laemmli buffer, run on a 12% polyacrylamide gel and detected with Coomassie brilliant blue staining. (2) protein molecular size standards (kDa). (B) Observation of the different complexes adsorbed on glutathione-Sepharose 4B beads under UV light. (C) Electron micrographs of ring-like structures produced by heterologous expression of N-GFP (left) and N (right) purified by GST-PCT. Bars, 50 nm. (D) N-GFP SRS (thin line) adsorption by RAW and D2SC/1 cell lines after 1 hour incubation at 4°C (controls: PBS in grey or GFP bold line). Green fluorescence associated with living cells was analyzed by flow cytometry (the gate was set up excluding dead cells stained with propidium iodide in FL3). The data (100,000 events) were acquired with a FACScalibur and analyzed with Cell Quest-Pro. (E) Confocal microscopy analysis showing entry of N-GFP SRS (green fluorescence) within cells. Filamentous actin was stained with phalloidin Rhodamin (red). Images of individual cells are either z sections through confocal images taken at sequential focal planes or xy views. Stacks of confocal images were acquired at 0.37 µm intervals (bars 10 µm).

In order to test whether N SRS complexes could target cells of the murine respiratory tract involved in antigen presentation, we studied the internalization by one macrophage and one dendritic cell line of fluorescent N SRS obtained by fusing the green-fluorescent-protein (GFP) at the C-terminus of N. The N-GFP chimeric protein was expressed in *E. coli* and purified according to the procedure previously described for N SRS [22]. The GST-PCT+N-GFP complex was purified to >95% homogeneity, as estimated by SDS-PAGE and Coomassie brilliant blue staining (Fig. 1A). Analysis of the purified complex adsorbed on glutathione-Sepharose beads under UV light revealed a strong green fluorescence for GST-GFP and GST-PCT+N-GFP, and no visible fluorescence for GST-PCT+N (Fig. 1B), indicating that the GFP fused at the C-terminus of N was correctly folded. To further characterize the PCT+N-GFP complex and determine whether it is constituted by SRS as it is the case for PCT+N, the samples were analyzed by negative-staining electron microscopy. Complexes containing N-GFP appeared mostly as ring-like structures with a less regular shape than SRS containing N only (Fig. 1C).

First, we used flow cytometry to check the capture of N-GFP SRS by murine macrophages (RAW) or dendritic cells (D2SC/1). As shown Fig. 1D, N-GFP SRS fluorescence was associated to cells after one hour of contact at 4°C. Only background level fluorescence was detected for cells incubated with soluble GFP. To demonstrate the internalization of N-GFP SRS by confocal microscopy, cells were incubated for one hour at 37°C with N-GFP SRS and then fixed and stained for filamentous actin with phalloidin-rhodamin (red). Punctuated green fluorescence was observed inside the cells for both cell lines (Fig. 1E). No signal was observed in cells incubated with soluble GFP (not shown).

Therefore, N SRS nanostructures are rapidly internalized by phagocytic antigen-presenting cells, presumably in endosomal compartments as suggested by the punctuated fluorescence.

### Nasal vaccination with N SRS protects against an RSV challenge

We investigated whether the delivery of N SRS nanostructures with the mucosal adjuvant LT(R192G) to adult BALB/c mice can induce protective immunity against a viral challenge with the human RSV-A2 strain.

**Figure 2:**
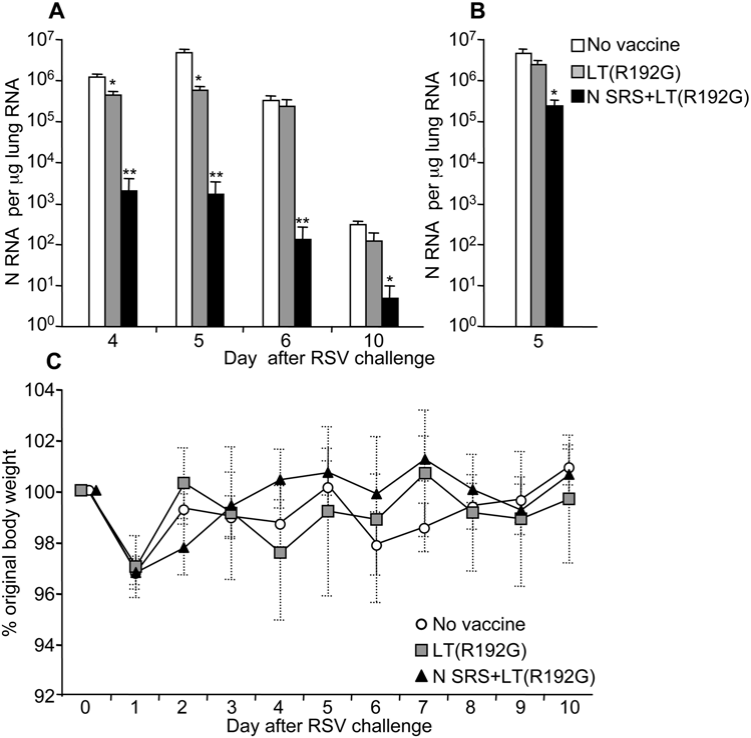
Nasal vaccination with N SRS protects against RSV replication in the lungs without causing disease exacerbation. BALB/c mice were administered twice at 2 weeks interval i.n. (A) or s.c (B) 10 µg N SRS and 5 µg LT(R192G) (black bars) or 5 µg LT(R192G) alone (grey bars). Two weeks after the second immunization, mice were challenged with 10^7^ PFU hRSV strain A2, together with a non-immunized control group of mice (white bars). Mice were monitored daily for body weight. Viral replication in lung was monitored by quantitative real-time RT-PCR. The number of N-specific RNA copies for 1 µg total reverse-transcribed lung RNA was determined against a standard curve using a plasmid encoding the viral N gene. (A) Intranasal immunization with N SRS reduced N-specific RNA in lungs 4 to 10 days after RSV challenge. (B) Sub-cutaneous vaccination induced moderate protection at 5 days after RSV challenge. Each bar represents the mean and SEM of 5–8 mice. (C) Weight loss in vaccinated versus non vaccinated groups is expressed as the percentage of initial weight (plot of mean±SEM, n = 4–5, day 0 is 100%). Data are representative from four independent experiments.

Non vaccinated (control) mice and mice administered twice intra-nasally (i.n.) LT(R192G) or N SRS+LT(R192G), 4 and 2 weeks before, were infected i.n. with RSV-A2 and killed from 4 to 10 days post-infection. A quantitative real-time RT-PCR, targeting viral N-RNA among total lung RNA was used to assess viral replication in lung tissue. As shown Fig. 2A, i.n. vaccination with N SRS+LT(R192G) (black bars) reduced very significantly the amount of N-specific RNA detected in lung RNA extracts (3-4 Log reduction at day 4, 5 and 6 p.i., p<0.001 and 2 Log reduction at day 10 p.i., p<0.05). A slight but transient decrease in N-RNA was also observed in mice administered twice the LT(R192G) adjuvant alone (grey bars, 1 Log reduction at day 4 and 5 p.i., p<0.05).

We also investigated whether sub-cutaneous administration of N SRS+LT(R192G) vaccine conferred any protection. Interestingly, mice vaccinated parenterally displayed only one Log reduction in their viral load at day 5 compared to non vaccinated animals (p<0.01) (Fig. 2B). Therefore the priming of local immune effectors by nasal vaccination seemed required in order to efficiently protect the mice against RSV replication.

Non vaccinated mice did not show any significant weight loss or illness from day 2 to day 10 after virus challenge (Fig. 2C), which is expected in a primary RSV infection [28]. Body weight loss is also an important parameter described in the literature for measuring the exacerbated illness caused by RSV vaccine based on the G or F envelope proteins [28]. Mice vaccinated i.n. with N SRS did not lose more weight than non vaccinated mice upon RSV challenge (Fig. 2C), thus showing that our vaccination strategy did not cause disease exacerbation upon virus infection.

### Nasal vaccination with N SRS triggers a mild inflammatory reaction in airways upon RSV infection

A major obstacle for vaccination against RSV is the risk of inducing an exacerbated lower respiratory tract disease due to an enhanced local inflammatory reaction following viral infection. Although mice vaccinated i.n. with N SRS and/or LT(R192G) did not show illness upon RSV challenge, we nevertheless investigated more closely for signs of inflammatory reaction in broncho-alveolar lavage (BAL) or lung tissue.

**Figure 3:**
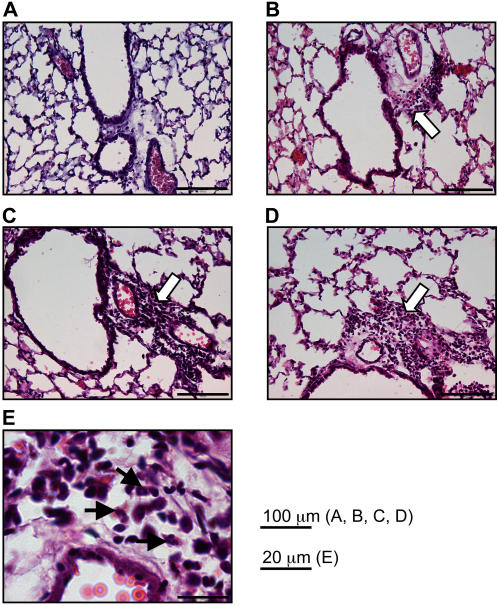
Nasal vaccination with N SRS and LT(R192G) augments cellular infiltration in lung tissue. BALB/c mice were administered i.n. 10 µg N SRS and/or 5 µg LT(R192G), twice at two weeks interval. Two weeks after the second immunization, all animals were challenged with 10^7^ PFU hRSV strain A2, together with a control group of non-immunized mice. One group of mice was neither immunized nor infected. Lung were dissected out 5 days post challenge, embedded in paraffin, sectionned at 7 µm and stained with eosin-hematoxylin. One representative section per group is shown (original magnification 20×, bars 100 µm). (A) control group (no vaccine, no virus), (B) primary infection group (no vaccine, RSV); (C) adjuvant only group (LT(R192G), RSV), (D) N SRS vaccinated group (N SRS+LT(R192G), RSV). Areas with an infiltration of inflammatory cells are indicated with a white arrow. (E) Enlargement showing that the immune infiltrate in N SRS vaccinated group was composed predominantly of lymphocytes and some neutrophils (black arrows) (original magnification 63x, bars 20 µm).

To assess pulmonary tissue infiltration, paraffin-embedded lung sections were stained with hematoxylin and eosin and carefully examined. Five days after RSV infection, lung sections from non vaccinated control mice showed diffuse peribronchiolar infiltrates (Fig. 3B, white arrow). Compared to these small lesions, lung sections from mice having received only the adjuvant (Fig. 3C) or the complete vaccinal preparation N SRS+LT(R192G) (Fig. 3D) displayed larger peribronchiolar and perivascular immune infiltrates (white arrows) mostly composed of lymphocytes. Few neutrophils were present within the lymphocytic infiltrates of lung sections from N SRS vaccinated mice (Fig. 3E, black arrows). No cellular infiltrate was found in the lung parenchyma, whatever the immunization treatment (Fig. 3). Thus N SRS vaccination caused a mild lung inflammatory reaction in contrast to the severe lung lesions and disease exacerbation reported in the literature for RSV vaccine based on the G surface protein [28].

**Figure 4:**
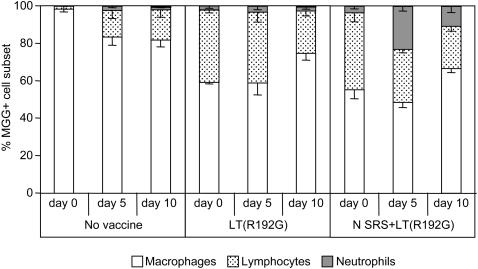
Nasal vaccination with N SRS induces neutrophil and lymphocyte recruitment in BAL. BALB/c mice were administered i.n. 10 µg N SRS and/or 5 µg LT(R192G) twice at two weeks interval. One group was not immunized. Two weeks after the second immunization, all animals were challenged with 10^7^ PFU hRSV strain A2. Cellular composition of BAL sampled at 0, 5 and 10 days after RSV challenge as determined by May-Grünwald-Giemsa staining: lymphocytes (dotted bars), macrophages (white bars) and neutrophils (grey bars). Results are expressed as mean and SEM percentages from 5–8 individual mice (Data from two experiments with similar results).

We also investigated which subsets of cells were recruited in the broncho-alveolar lumen following RSV infection of vaccinated mice. The total number of viable cells recovered from the BAL at 5 days post challenge was markedly enhanced for LT(R192G) and even more for N SRS+LT(R192G) vaccinated mice (Table 1). After cytocentrifugation and May-Grünwald-Giemsa staining of BAL cells collected at days 0, 5 and 10 post RSV challenge (Fig. 4), we observed that 2 administrations of LT(R192G) alone or N SRS+LT(R192G) had increased significantly the percentages of lymphocytes in BAL compared to the control non vaccinated group (Fig. 4 and Table 1). Moreover, virus challenge of mice vaccinated with N SRS+LT(R192G) resulted in an increased percentage of neutrophils in BAL compared to non vaccinated mice or mice treated with LT(R192G) only (Fig. 4 and Table 1). Yet, at no time point was there any significant presence of eosinophils in the BAL of any group.

**Table 1:** Inflammatory cellular infiltrate in BAL, 5 days after RSV challenge

	Number of cells (×10^4^) (a)	% lymphocytes (b)	% neutrophils (b)
^(1)^ No vaccine	3.7±0.7 (n = 5)	14.3±4.3 (n = 8)	2.2±0.7 (n = 8)
^(2)^ LT(R192G)	8.3±1.5 (n = 4)	37.9±5.4 (n = 8)	3.1±1.8 (n = 8)
^(3)^ N SRS+LT(R192G)	15.5±3.6 (n = 4)	28.4±1.8 (n = 7)	22.9±2.5 (n = 7)
*p* (U Mann-Whitney)	^(1)^ versus ^(2)^ <0.05	^(1)^ versus ^(2)^ <0.01	^(1)^ versus ^(2)^ ns
	^(1)^ versus ^(3)^ <0.05	^(1)^ versus ^(3)^ <0.05	^(1)^ versus ^(3)^ <0.001
	^(2)^ versus ^(3)^ ns	^(2)^ versus ^(3)^ ns	^(2)^ versus ^(3)^ <0.001

(a) viable cells were counted after tripan blue exclusion of dead cells.

(b) % was determined after May-Grünwald-Giemsa coloration.

### Nasal vaccination with N SRS generates antigen-specific CD4^+^ and CD8^+^ T cell in spleen and regional lymph nodes

The nucleocapsid protein when expressed by recombinant vaccinia virus is known to be an important target for cellular responses, both in mice [15] and calves [12]. We thus wondered whether N SRS would generate antigen-specific CD4^+^ and/or CD8^+^ T responses in mice.

**Figure 5:**
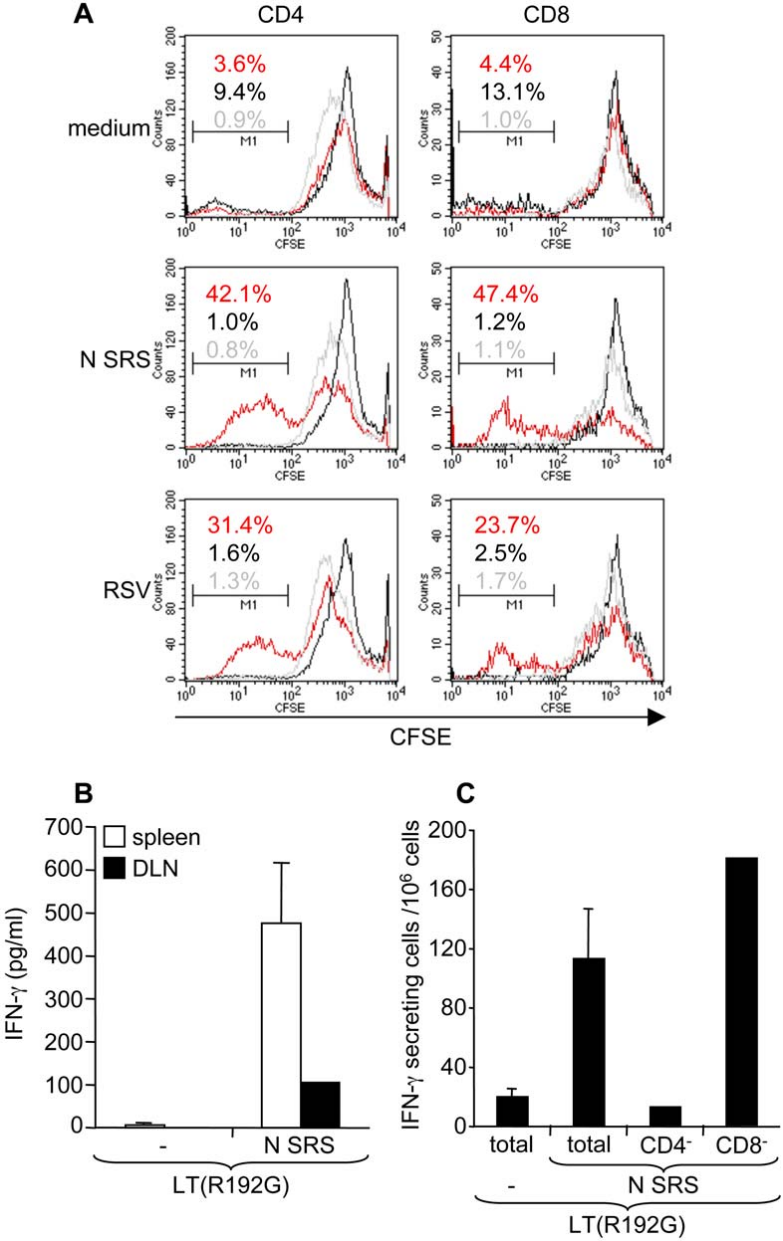
T cell-mediated immune response to N SRS. Nasal vaccination with N SRS generated antigen-specific CD8^+^ T cells and IFN-γ producing CD4^+^ T cells. (A) Antigen-specific proliferation of CD4^+^ and CD8^+^ T splenocytes after 7 days restimulation with RSV-A2 (1 PFU/cell), N (10 µg/ml) or medium. Pooled splenocytes from non immunized (grey lines), LT(R192G) (black lines) or N SRS+LT(R192G) (red lines) immunized groups were stained with CFSE, cultured for 7 days and then labeled with anti-CD8-biot and anti-CD4-PE for flow-cytometry analysis. The data (100,000 events) were acquired with a FACScalibur and analyzed with Cell Quest-Pro. The CD8^+^ or CD4^+^ lymphocyte population was gated according to SSC/FCS and FL4 (CD8) or FL2 (CD4) fluorescence criteria and the fluorescence corresponding to CFSE was monitored in FL1. The percentage of proliferating cells (low CFSE staining) is indicated on the plot with the color corresponding to the immunization condition. (Data from one out of two experiments with similar results). (B) Two weeks after the booster immunization with LT(R192G) or N SRS+LT(R192G), spleen (white bars) and draining LN (cervical and sub-maxilliary LN, black bars) were dissected out and cell suspensions prepared. Splenocytes from individual mice and pooled draining LN cells from each group of mice were re-stimulated for 72 hr with N SRS (10 µg/ml) or medium (mock). IFN-γ secretion was measured in cell culture supernatant with a standardized specific sandwich ELISA assay (white bars represent the mean and SEM of 5 individual spleens, black bars represent the pool of LN, data from one out of three experiments). (C) The frequency of IFN-γ secreting splenocytes after 20 hr restimulation with N (10 µg/ml) was monitored by ELISPOT. Spleen cells from LT(R192G) or N SRS+LT(R192G) immunized mice were assayed for each mouse (each bar represents the mean and SEM of 5 mice). Depletion of CD4^+^ or CD8^+^ T cells was done by immuno-magnetic separation of pooled splenocytes from either LT(R192G) or N SRS+LT(R192G) groups. (Data from one out of two experiments with similar results).

CFSE-stained splenocytes from non vaccinated, LT(R192G) or N SRS+LT(R192G) immunized mice were co-cultured with either live RSV, N SRS or medium alone and after 7 days the level of CFSE staining on CD4^+^ and CD8^+^ cell subsets was quantified by flow cytometry analysis (Fig. 5A). A large percentage of CD4^+^ and CD8^+^ splenocytes isolated from N SRS immunized mice and co-cultured with N SRS or RSV displayed low CFSE fluorescence, showing the expansion of CD4^+^ and CD8^+^ N-specific T cells (Fig. 5A red lines). To control for the specificity of this response, we showed that CD4^+^ and CD8^+^ splenocytes isolated from non immunized mice (grey lines) or LT(R192G) treated mice (black lines) did not proliferate in response to either RSV or N SRS (Fig. 5A).

To get further insights into the function of these antigen-specific CD4^+^ and CD8^+^ T cells, splenocytes from individual mice or pooled draining lymph node cells were re-stimulated *in vitro* for 72 hr with N (10 µg/ml) or medium (negative control). IFN-**γ** was assayed in cell culture supernatants by specific sandwich ELISA. Two nasal administrations of N SRS and LT(R192G), two weeks apart, elicited memory Ag-specific IFN-γ producing cells in spleen and to a lesser level in the respiratory tract-draining lymph node (LN) (Fig. 5B). Besides, we could not detect IL-5 nor IL-10 in the same cell culture supernatant, whereas these cytokines were readily detected in PMA and ionomycine stimulated spleen or lymph node cell supernatants (data not shown).

In order to distinguish between CD4^+^ and CD8^+^ T cells as IFN-γ sources, splenocytes were depleted in either subset by magnetic-beads separation. The frequency of Ag-specific IFN-γ producing cells in total, CD4^+^ or CD8^+^ depleted cell populations was monitored by ELISPOT upon 20 hr co-culture with N SRS. We found that IFN-γ producing cells were nearly absent from CD4^+^-depleted splenocytes and that their frequency was slightly increased in CD8^+^-depleted splenocytes (Fig. 5C). These data clearly demonstrate that N SRS nasal vaccination primed antigen-specific IFN-γ producing CD4^+^ T cells.

Thus we can conclude that N SRS mucosal immunization elicited both CD4^+^ and CD8^+^ antigen-specific memory responses, with a Th1 cytokine secretion pattern.

### Nasal vaccination with N SRS followed by RSV challenge results in the presence of activated CD4^+^ and CD8^+^ lymphocytes in airways

We then wondered if the memory N-specific CD4^+^ and CD8^+^ T cells induced by vaccination would generate effector cells at the site of virus replication, that is the airways. To answer this question, BAL cells collected before and 4–10 days post infection were stained with anti-CD69 (early activation marker) in combination with either anti-CD4 or anti-CD8 and analyzed by flow cytometry.

**Figure 6:**
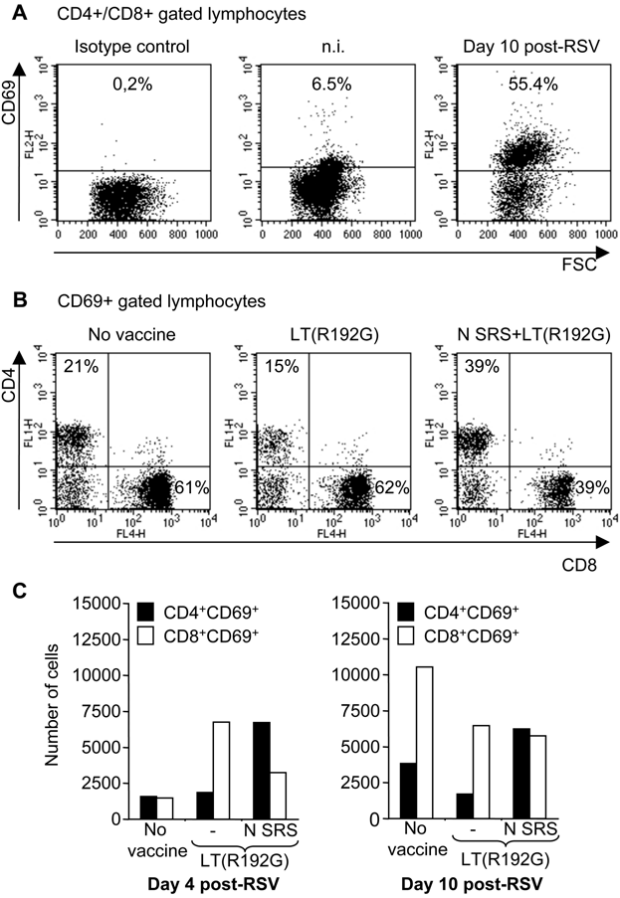
Nasal vaccination with N SRS followed by virus infection triggers CD69^+^CD4^+^ and CD69^+^CD8^+^ lymphocytes recruitment in BAL. BALB/c mice were administered i.n. twice at 2 weeks interval 10 µg N SRS and/or 5 µg LT(R192G). Two weeks after the second immunization, N SRS+LT(R192G) or LT(R192G) treated mice and a control group of non-immunized mice were challenged with 10^7^ PFU hRSV strain A2. BAL cells were collected from non-infected vaccinated mice and at days 4 and 10 post-RSV challenge of vaccinated and control mice. Live cells were numerated after trypan blue exclusion. Cells (pool of 5 mice per group) were stained with fluorochrome-conjugated Abs anti-CD4, -CD8 and -CD69 and analyzed on a FACS Calibur, collecting data on at least 5,000 lymphocytes gated according to FSC and SSC criteria. (A) Gated on CD4^+^/CD8^+^ lymphocytes. First plot shows matched-isotype background staining for anti-CD69 (pool of BAL, 10 days post-RSV). Second and third dot plots show CD69 staining among BAL cells from N SRS+LT(R192G) vaccinated mice, respectively non infected (n.i.) or 10 days after RSV infection. The % of CD69^+^ cells is indicated on the plots. (B) Gated on CD69^+^ lymphocytes. Percentage of CD4^+^ and CD8^+^ cells, 10 days after virus challenge of non vaccinated mice (first plot), LT(R192G) or N SRS+LT(R192G) immunized mice (second and third plots, respectively). The % of CD4^+^ and CD8^+^ cells are indicated on the plots. (C) The total number of CD4^+^CD69^+^ (black bars) and CD8^+^CD69^+^ (white bars) lymphocytes in BAL was calculated as (% positively stained cells)×(% gated cells)×(number of live cells) at 4 and 10 days after RSV infection.

The CD8^+^ and CD4^+^ lymphocytes recovered in BAL from non infected mice, two weeks after booster immunization with LT(R192G) or N SRS+LT(R192G) were mostly CD69^−^ (Fig. 6A, n.i.) and expressed high levels of CD44 (data not shown), such phenotype corresponding to memory cells. RSV infection of either non vaccinated, LT(R192G) or N SRS+LT(R192G) vaccinated mice induced a massive activation of BAL CD4^+^ or CD8^+^ lymphocyte subsets, with CD69 being expressed on more than 50% of the gated cells (Fig. 6A, day 10). The proportion of CD4^+^ and CD8^+^ cells among CD69^+^ lymphocytes is shown (Fig. 6B) 10 days after virus infection. Interestingly activated CD69^+^ BAL lymphocytes from N SRS vaccinated mice were equally composed of CD8^+^ and CD4^+^ cells whereas CD69^+^ BAL lymphocytes from non vaccinated or LT(R192G) immunized mice were mostly CD8^+^ (Fig. 6B). The total number of CD4^+^CD69^+^ or CD8^+^CD69^+^ lymphocytes in BAL 4 and 10 days after RSV challenge is shown (Fig. 6C). Intra-nasal immunization with N SRS was specifically associated with the presence of CD4^+^CD69^+^ lymphocytes as early as 4 days and up to 10 days after virus infection (Fig. 6C), as compared with non vaccinated or LT(R192G) immunized mice where CD8^+^CD69^+^ lymphocytes were the predominant subset (Fig. 6C).

Thus, virus challenge of mice vaccinated intra-nasally with N SRS+LT(R192G) was characterized by a balanced infiltration of CD8^+^CD69^+^ and CD4^+^CD69^+^ lymphocytes in the airways, among which the CD4^+^CD69^+^ subset presumably reflected the priming of N-specific CD4^+^ T cells upon vaccination.

### Nasal vaccination with N SRS elicits systemic and mucosal N-specific IgG1, IgG2a and IgA antibodies

**Figure 7:**
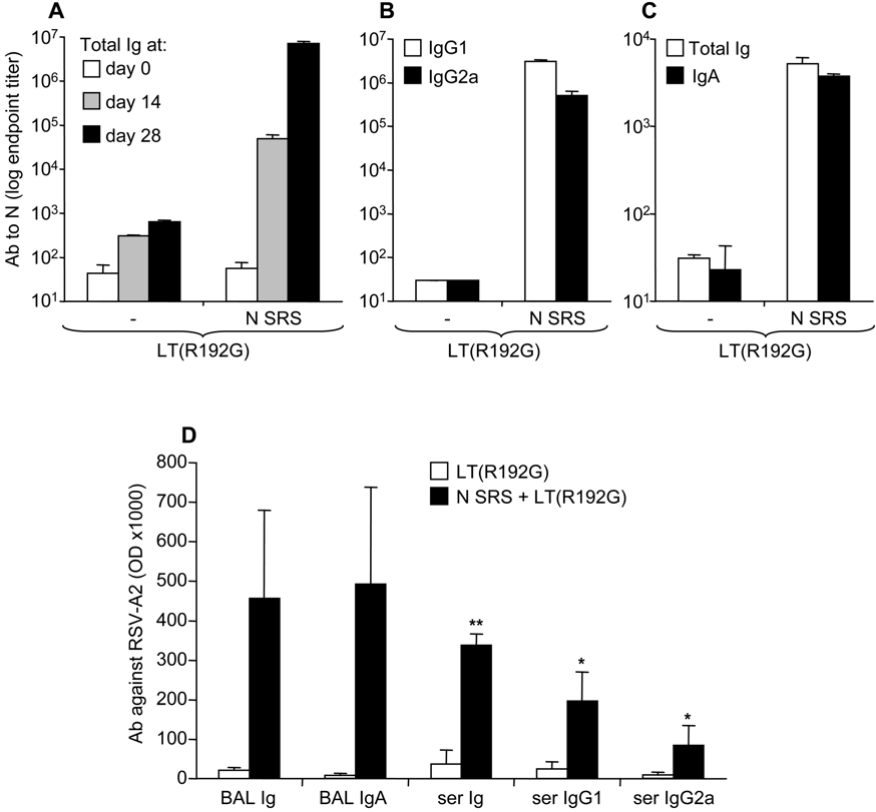
Systemic and mucosal antibody response to N SRS administered intra-nasally. BALB/c mice were administred 10 µg N SRS and/or 5 µg LT(R192G) twice i.n. at two weeks interval. Individual sera were collected at day 0, 14 and 28 and individual BAL supernatants at day 28. (A) Total Ig, (B) IgG1 (white bars) and IgG2a (black bars) anti N antibodies in serum. (C) total Ig (white bars) and IgA (black bars) in BAL. Titers were determined in an endpoint dilution ELISA assay on plates coated with N SRS. Each bar represents the mean and SEM of 5–6 mice. Titers are shown with logarithmic scale (data from one out of three experiments with similar results). (D) Systemic and mucosal antibodies raised against N SRS recognize RSV-A2. Total Ig, IgG1, IgG2a and IgA in BAL or serum were titrated by ELISA on plates coated with RSV infected HEp-2 versus non infected HEp-2 cells. Data shown are ODx1000 at dilution 270 for sera samples and dilution 27 for BAL samples, after subtracting OD value of non infected HEp2. Each bar represents the mean and SEM of 5–6 mice (Data from one out of two experiments with similar results).

Intranasal administration of N SRS and LT(R192G) to adult BALB/c mice induced N-specific antibody responses, as assayed by ELISA in serum and BAL samples taken 2 weeks after the primary and secondary immunization (Fig. 7). A single immunization with N SRS and adjuvant resulted in significant production of anti-N Ab in serum and a secondary type of response was detected after a second i.n. administration two weeks later (Fig. 7A: day 0 versus day 14 and day 14 versus day 28 p<0.05, n = 5). N SRS immunization elicited both IgG1 and IgG2a serum antibodies, with IgG1 titers being 5 times higher than IgG2a titers two weeks after the boost (p<0.01 Fig. 7B), thus suggesting a mixed Th1/Th2 type of immune response. Finally two successive nasal immunizations with N SRS also elicited strong local Ig responses dominated by the IgA isotype in BAL (Fig. 7C). Sub-cutaneous administration of N SRS and LT(R192G), two weeks apart, elicited similar levels and pattern of antibody responses in serum but failed to prime for anti-N IgA in BAL (data not shown). Systemic and mucosal antibodies elicited against N SRS were also able to recognize the native viral N as shown by their specific binding to RSV-infected HEp2 cells (Fig. 7D). We tested whether serum and BAL N-specific antibodies had the capacity to neutralize RSV-A2 replication *in vitro* on HEp-2 monolayers with or without 2% guinea-pig complement (Sigma). Negative controls were serum and BAL Ig harvested from non-immunized mice and the positive control was goat anti-RSV antibodies (Serotec). We found no evidence of virus neutralization by serum and BAL antibodies to N as compared to positive and negative controls (data not shown). It is thus unlikely that the antibody response primed upon N SRS vaccination will play a direct role in the protection against virus infection.

## Discussion

Early attempts at vaccinating children against RSV infection using a formalin inactivated virus resulted in a dramatic aggravation of disease, linked to the exacerbation of inflammatory reactions in the lower respiratory tract. Hence, despite more than 40 years of intense research, there is still no vaccine licensed against human RSV, although some live attenuated RSV vaccines obtained by reverse genetic have now reached the stage of clinical trials in infant cohorts [29]. However, in a context where even wild type RSV infections fail to induce a strong long lasting protective immunity, these recombinant RSV vaccines, which can be less immunogenic than the wild type virus because of attenuation, may not bring the final clue to the issue of RSV vaccination. Conversely subunit vaccines have the advantage of safety, but need to be formulated and delivered appropriately in order to induce the desired magnitude and quality of immune responses. Ideally a vaccine against RSV should prevent virus dissemination from the upper to the lower airways, thus reducing the intensity of the inflammatory detrimental immune response in the lungs.

We are the first to propose an efficient vaccination strategy against RSV using the nucleocapsid protein alone as a vaccine antigen, under the form of soluble nanoparticles composed of 10–11 N units assembled around a bacterial RNA (referred to as N SRS). In the present study we have demonstrated that N SRS are highly immunogenic when delivered via the nasal route together with LT(R192G) and that the immune response primed upon vaccination is protective against an RSV challenge. Work is in progress to show the immunogenicity of N SRS in newborn calves and mice, that is at the period of life when the immune system is considered to respond poorly to vaccination [30].

The strong immunogenicity of N SRS nanoparticles could be due to their highly organized and repeated structure, as was reported for the core proteins of the human hepatitis B virus [31], the Measles virus [21], and the Newcastle disease virus [32]. Moreover we demonstrated in the present study that N SRS were rapidly taken up by dendritic cell and macrophage cell lines representing key subsets for the induction of immune responses in the respiratory tract. These observations are in agreement with the findings by others demonstrating that the nucleocapsid protein of measles virus binds to different cells types (dendritic cells, monocytes and epithelial cells,) and that at least two receptors are implicated in this process: FcγRII/III and a second one not yet identified [33]. We are now investigating the mechanisms by which N SRS are taken up by macrophage or dendritic cells, whether or not N SRS internalization involves a specific receptor and what are the consequences of N SRS engulfment by airway dendritic cells in term of maturation and pathway of antigen presentation. Potentially, once N SRS are internalized and processed by dendritic cells, the bacterial RNA they contain, could be made accessible for cellular receptors such as TLR7 or TLR8 and provide the signaling for maturation [34].

The most important finding of our study was to demonstrate that two successive nasal immunizations with N SRS and LT(R192G) at 2 weeks interval, drastically reduced RSV replication in the lungs, without causing disease exacerbation. However, an enhanced inflammatory response to virus infection was recorded in the airways, with an infiltration of lymphocytes and neutrophils in the BAL and in the peribronchovascular spaces.

We also showed that sub-cutaneous delivery of N SRS and LT(R192G) was far less efficient at stimulating protective immunity against RSV than the intra-nasal delivery. Thus we can conclude that a mucosal route of antigen delivery is required in order to prime local immune effectors. As CTL directed against N epitopes were shown to be protective against RSV infection in cattle and mice [17], [18], we anticipated that nasal vaccination with N SRS would elicit antigen-specific memory CD8^+^ T lymphocytes. We have indeed recorded a memory CD8^+^ T cell response in spleen following immunization with N SRS. N SRS immunization also induced antigen-specific IFN-γ producing CD4^+^ memory cells in spleen and regional lymph nodes. Upon viral challenge of vaccinated mice, effector CD4^+^ and CD8^+^ lymphocytes were rapidly recruited to the BAL. Finally, nasal immunization with N SRS generated strong local and systemic Ab responses, including a massive anti-N IgA Ab production in BAL. However these antibodies did not neutralize virus replication *in vitro*.

We can draw several hypothesis from these observations about the role of the immune effectors primed by N SRS nasal vaccination in the viral clearance.

First, a previous report with another modified LT (LTK63) delivered alone intranasally demonstrates its capacity to reduce the inflammatory lesions caused in lung tissue by respiratory virus infections [35]. This phenomenon, called innate imprinting, reduces as well virus replication in lungs following Influenza or RSV infection [35]. Therefore LT(R192G) may have contributed by similar mechanisms to the partial protection we observed against RSV replication. However, in our experimental conditions, LT(R192G) triggered an increased CD8^+^ lymphocytic infiltration of the airways upon virus challenge. Whether this priming for CD8^+^ cells is beneficial or detrimental to the outcome of virus-induced pathology needs to be elucidated. Of note, in our preliminary vaccination trials, N SRS administered without LT(R192G) failed to protect mice against virus replication.

Second, CD4^+^ and CD8^+^ T lymphocytes play a major role in the immune responses to respiratory virus infections (ex influenza and parainfluenza viruses) [36]. Functional memory CD4^+^ T cells were shown to persist in the lung following respiratory virus infection and then provide a substantial degree of protection against secondary infection [37]. This protection is mediated at least in part by IFN-γ and does not depend on antibody [38]. Interestingly IFN-γ secreting CD4^+^ T cells have already been shown to be the major protective element against RSV in trials using the sub-unit vaccine BBG2Na [39].

On the other hand, although the protective role of CD8^+^ T cells against RSV infection is well documented [9], they can provoke lung-disease exacerbation when in excess, as reported in vaccination assays with the dominant K^d^ restricted CTL epitope from the M2 protein of RSV [40], [41]. Conversely, the production of IFN-γ by CD4^+^ or CD8^+^ T cells down-regulated the pathological immune mediators in murine experimental models of vaccine-mediated lung-disease exacerbation [10], [42]. Thus, a moderate CD8^+^ T cell priming and a strong Th1 imprinting might be a clue to why we obtained protection against RSV challenge with mild inflammation at the lung tissue level.

Third, secretory IgA antibodies play a central role in protecting mucosal surfaces against infections by blocking the entry of pathogens into target epithelial cells and/or by forming immune complexes that will be trapped into mucus, a defense mechanism referred to as immune exclusion [43]. Since N is not expressed at the surface of RSV virions, in contrast to the fusion (F) or attachement (G) protein, anti-N antibodies are not likely to neutralize RSV via immune exclusion. Indeed anti-N antibodies generated after vaccination with recombinant vaccinia virus expressing N or after vaccination with N SRS were not neutralizing ([44] and our data).

Intra-cellular neutralization might be another way for IgA to neutralize viruses as recently proposed at least *in vitro*: IgA antibodies against the inner protein VP6 of rotavirus neutralize viral replication in the endosomal compartment of target epithelial cell [45] and IgA directed against measles virus N efficiently block virus replication in epithelial cells [46]. Thus it can be hypothesized that the anti-N IgA responses, triggered by nasal vaccination with N SRS, could limit RSV dissemination by interfering with its replication inside the cells from the respiratory tract epithelium. However preliminary passive transfert experiments, either intranasally or intra-peritoneally, with Ig purified from BAL and serum of N SRS vaccinated mice, did not confer any significant protection against virus replication.

Finally, the early infiltration of neutrophils that we observed in BAL upon RSV challenge of vaccinated mice could also participate to virus clearance. Indeed neutrophils have recently been described to be implicated in RSV clearance [47].

In the present study we have successfully evaluated a new subunit vaccination strategy based on intra-nasal delivery of nanometric sub-nucleocapsid ring structures of RSV. Further investigations will be required to decipher the exact mechanisms of the protection we have observed. Knowing the exact pathways by which a vaccination strategy against RSV is efficient will also help to minimize the risk of disease exacerbation in the target population, that is newborn children or newborn calves.

## Materials and Methods

### Plasmid constructions

The pGEX-P and pET-N plasmids which contain sequences from the RSV Long strain [48] have been described previously [49]. For expression of GFP in *E. coli*, the EGFP gene was subcloned from pEGFP-N1 (Clontech) at the *Sma*I-*Nco*I sites in pGEX-4T3 (Amersham Biosciences). A pET-N-Sac was derived from pET-N by introducing a *Sac*I restriction site at the end of the N-coding sequence by using the Pfu DNA polymerase with the QuickChange Site-Directed Mutagenesis Kit (Stratagene) according to the manufacturer's instructions (sequence of primers available on demand). This mutation (GAG CTT–>GAG CTC) did not modify the amino acid sequence of the two last residues of N. To insert the EGFP coding sequence in frame at the end of N, plasmid pET-N was digested by *Eag*I, blunt ended by DNA polymerase Klenow fragment, and digested by *Sac*I. Plasmid pEGFP-N1 (Clontech) was digested by *Eag*I, treated with DNA polymerase I Klenow fragment, digested by *Sac*I, and the EGFP coding sequence was inserted into pET-N. The resulting plasmid was designated pET-N-GFP, and encodes for a N-GFP fusion protein.

The integrity of all constructs was assessed by DNA sequencing.

### Expression and purification of recombinant proteins from *E. coli*

*E. coli* BL21 (DE3) (Novagen) cells were co-transformed with either the plasmids pGEX-P and pET-N or the plasmids pGEX-P and pET-N-GFP. Recombinant protein expression and purification of the GST-fusion proteins was performed with glutathione-Sepharose 4B beads (Pharmacia) as previously described [22]. This protocol allows the purification of recombinant N and N-GFP proteins via their capacity to interact with the C-terminal fragment of P (residues 161–241) fused to GST (named GST-PCT). N+PCT and N-GFP+PCT were separated from the beads by thrombin cleavage as previously described [22]. N and N-GFP were further purified by gel filtration in order to remove the PCT fragments [22].

### Electron microscopy

Samples containing purified N and N-GFP proteins were applied to an air-glow-discharged carbon-coated grid and stained with a 2% uranyl acetate aqueous solution. Grids were observed in a CM12 electron microscope (Philips) operated at 80 kV. Micrographs were recorded at a nominal magnification of 35000 on Kodak SO163 Electron Plate, developed 12 min in Kodak D19. The scanned micrographs were visualized with Photoshop (Adobe Systems).

### Cell lines

The RAW264.7 murine macrophage cell line (given by Dr. H Bierne, Institut Pasteur, France) was grown in DMEM (Sigma) supplemented with 10% fetal calf serum (FCS), 2mM L-glutamine, 100U/mL Penicillin and 0.1mg/mL Streptomycin. The D2SC/1 murine splenic dendritic cell line (given by Prof. P. Ricciardi-Castagnoli, University of Milano, Italy) was grown in Iscove's Modified Dulbecco's medium (Sigma) supplemented with 5% FCS, 2mM L-glutamine, 100U/mL Penicillin, 0.1mg/mL Streptomycin (PS) and 50 µM β-Mercaptoethanol.

### Flow cytometry and confocal microscopy

The N-GFP and GFP were quantified on a Coomassie blue-stained SDS-PAGE using ImageJ software and a dilution range of BSA as standards. RAW and D2SC/1 cells were incubated in D-PBS (Gibco) supplemented with 5 mM EDTA, 0.33% lidocaine (Astra Zeneca) for 2 min at 37°C and centrifuged for 7 min at 500 *g.* The pellet was resuspended in PBS supplemented with 2% FCS. Viable cells were adjusted to 10^7^ cells/mL and incubated in round bottom tubes with 30 µM GFP or N-GFP SRS for one hour at 37°C together with propidium iodide at a final concentration of 1 µg/ml. The association of GFP with cells was then analyzed by flow cytometry using a FACS Calibur (Becton Dickinson). Data analysis was performed on 50,000 events with Cell Quest Pro software (Becton Dickinson). Dead cells were excluded on the basis of propidium iodide labeling.

For confocal microscopy examination, cells treated following the same protocol but without addition of PI, were fixed, permeabilized using the “Fix and Perm” kit (Caltag) according to the manufacturer instructions, and stained with 0.4 U/mL phalloidin-rhodamin (Molecular Probes). After 15 min incubation, cells were resuspended in PBS supplemented with 2% FCS, and 10^5^ cells were centrifuged (Cytospin 2, Chandons) on Superfrost plus slides (SFPLUS-42, Milian) and mounted in Fluoromount G (InterBio Tech). Dual-immunofluorescence confocal images were acquired with a confocal laser scanning microscope (LSM 510 META, Zeiss) equiped with a Plan-Achromat 63× oil immersion objective (1.4 numerical aperture). Stacks of confocal images were acquired at 0.37 µm intervals. Images were processed using Zeiss LS Image Browser software for overlays of red and green channels.

### Vaccine formulation and virus

The detoxified *E. coli* enterotoxin LT(R192G) was provided endotoxin free by Prof. Clements (Tulane University, New Orleans, USA). Purified N SRS had an endotoxin content of 1–3 EU/mg protein (QCL-1000 LAL assay, Cambrex).

The RSV strain A2 (provided by Prof. Openshaw, Imperial College, Saint Mary's Hospital, London) was propagated and titrated by plaque assay on HEp-2 monolayers.

### Immunization of Mice

Eight- to 10-week-old BALB/c female mice were purchased from Charles River Laboratories (Lyon, France) and bred under specific pathogen-free conditions in the INRA animal care facilities (Jouy-en-Josas, France). All animal experiments were carried out under the authority of licence issued by the Direction des Services Vétérinaires (accreditation number 78-27).

Anaesthetized mice were immunized intra-nasally twice, two weeks apart, with 10 µg N SRS and/or 5 µg LT(R192G) in 50 µL 0.9% apyrogen NaCl. In some experiment mice were vaccinated sub-cutaneously. For viral challenge, mice were infected intra-nasally with 10^7^ PFU RSV-A2, two weeks after the booster immunization. Their weight was monitored daily up to euthanasia. Sera were obtained from blood collected via retro-orbital punction at day 0, 14 and 28 post-immunization or just before killing by cervical dislocation at 4–10 days post-infection. Lymph nodes draining the upper respiratory tract (cervical and maxilliary LN) and spleen were dissected out. The bronchus connected to the left lobe of the lung was clamped. The left lobe of the lung was snap frozen in liquid nitrogen and kept frozen at −80°C until extraction of RNA for RT-PCR. To collect broncho-alveolar lavage fluids (BAL), the mouse trachea was surgically exposed, cannulated with a syringe and the remaining lobes of the lungs were flushed four times in and out using 1.5 mL D-PBS (Gibco) supplemented with 1mM EDTA (Gibco). After centrifugation (4 min, 500g), viable BAL cells were resuspended at appropriate concentration for cytocentrifugation. Sera and BAL supernatants were stored frozen at −20°C. For histological analysis, the lungs were inflated via the trachea with 1 mL of PBS with 4% paraformaldehyde and 2% saccharose, dissected out and immerged into PBS, 4% PFA and 2% saccharose for a week, then dehydrated and embedded in paraffin.

### Determination of pulmonary RSV load by Real-time PCR

Frozen lungs were homogenized directly in lysis buffer (Absolutely RNA miniprep kit, Stratagene) using RNAse free microtubes and pellet pestle (Kimble-Kontes). Total lung RNA was extracted using the Absolutely RNA miniprep kit (Stratagene). Total RNA amount was quantified at 260 nm. Of each RNA sample, 1 µg were reverse transcribed for one hour at 42°C, using 300 U of M-MLV Reverse Transcriptase (SuperScript II, Invitrogene) with 7 µM of random hexanucleotide primers (pd(N)_6_, Pharmacia Biotech), 15 nmol of each dNTP, 5 mM DTT and 60 U of ribonuclease inhibitor (RNAseOUT, Invitrogen). The quality and relative amount of cDNA was assessed by semi-quantitative PCR for β-actine as previously described [50].

Quantitative real time-PCR was performed targeting the conserved region of the RSV-N gene as described by Chavez-Bueno [51]. Briefly, a 84 bp amplification product corresponding to positions 42 to 125 of the N gene from the hRSV-A2 strain was obtained using the AGATCAACTTCTGTCATCCAGCAA upstream and the TTCTGCACATCATAATTAGGAGTATCAAT downstream primers. Q-PCR was run on a Perkin Elmer ABI Prism 7700 Sequence Detector, using the SYBR GREEN PCR Master Mix (Applied Biosystems) and 300 nM of each primer. cDNA samples were run in triplicate (111 ng per reaction) as well as non template controls. In each assay, serial ten fold dilutions of the plasmid pET-N were run in duplicate, allowing to quantify the number of N gene copies generated from unknown samples by comparison of the cycle threshold values using the SDS 1.9.1 software. Results were expressed as the number of copies of N RNA per µg of input total lung RNA. The detection limit of the PCR method was calculated according to the cycle threshold values of non-infected control lungs and the highest dilution of pET-N giving the same cycle threshold value. We estimated the detection limit of our assay to be 20 copies of N per µg of input total lung RNA.

### May-Grünwald-Giemsa staining and histology

Macrophages, lymphocytes, neutrophils and eosinophils were enumerated by microscopic examination of May-Grünwald and Giemsa stained cytocentrifuge slides. At least 300 cells/sample were counted.

Lung sections (7 µm) were prepared from paraffin-embedded tissue using a microtome and stained with hematoxylin and eosin.

### Flow cytometry analysis of BAL lymphocytes

After a first 20 min incubation with anti-CD32/CD16 (FcBlock, BD bioscience), BAL cells were stained with anti-CD4-FITC, anti-CD8-Biot and anti-CD69-PE conjugated rat anti-mouse Abs (BD bioscience) for 30 min on ice, washed and then incubated for another 30 min on ice with streptavidin-APC (BD bioscience). Cells were then fixed in 10% CellFIX (BD bioscience). All samples were analyzed on a FACScalibur (BD Biosciences) collecting data on at least 5,000 lymphocytes gated according to their forward and side scatter features.

### CFSE staining of splenocytes and CD8/CD4-proliferation assay

Freshly isolated, red blood cells depleted splenocytes were incubated with 2 µM CFSE (Molecular Probes) in RPMI without FCS for 10 min at 37°C and then co-cultured for 7 days as described above (section 4.9) with either N SRS (10 µg/mL final concentration), hRSV-A2 (1 PFU/cell) or with medium alone as negative control all diluted in RPMI 1640 supplemented with 10% FCS, PS, 2mM L-glutamine and 50 µM β2-mercaptoethanol (Sigma). After a first 20 min incubation with anti-CD32/CD16 (FcBlock, BD bioscience), CFSE-labeled splenocytes were stained with an anti-CD8-Biot and with an anti-CD4-PE (BD bioscience) for 30 min on ice, washed and then incubated for another 30 min on ice with streptavidin-APC (BD bioscience). Cells were then fixed in 10% CellFIX (BD bioscience) and analyzed on a FACScalibur (BD Biosciences) collecting 100,000 events. Proliferating cells were characterized as CFSE^low^. As expected CFSE^low^ lymphocytes displayed higher FSC and SSC parameters than their non-dividing CFSE^high^ counterpart.

### IFN-γ production by immune spleen or lymph node cells

Individual spleens and pooled lymph nodes were minced and mashed gently through a 100 µm nylon filter (Falcon). Red blood cells depleted splenocytes and lymph node cells were adjusted to 2.10^6^ viable cells/mL in RPMI 1640 supplemented with 10% FCS, PS and 2 mM L-glutamine and 4.10^5^ cells/well were distributed in 96-well flat-bottomed microtiter plates (Falcon 3072). Cells were then incubated in triplicates with either N SRS (10 µg/mL final concentration) or, as negative control, with medium alone or, as positive control, with 10 ng/mL PMA (Phorbol 12-myristate 13-acetate, Sigma) and 1 µg/mL Ionomycin (Calbiochem). Cell cultures were incubated at 37°C in 5% CO_2_ for 72 hr, and supernatants were harvested and kept frozen at −20°C until assayed.

Supernatants were assayed for IFN-γ by a standardized ELISA using capture MAb R4-6A2 and biotinylated XMG1.2 (BD biosciences) as previously described [52]. Murine rIFN-γ (R&D systems) diluted from 3300 to 1.5 pg/mL was added in duplicate wells to establish a standard curve.

### Immuno-magnetic depletion of CD4^+^ and CD8^+^ lymphocytes

Splenocytes (10^7^ cells) resuspended in D-PBS (Gibco), 1mM EDTA and 2% FCS, were incubated with 5 µg/mL anti-CD16/CD32 (FcBlock, BD bioscience), and 1 µg/mL FITC-conjugated anti-CD4 or anti-CD8α (BD bioscience) for 15 min on ice with gentle agitation. Cells were washed twice, incubated for 15 min with the EasySep FITC-selection cocktail and for a further 10 min with the EasySep magnetic particles before cell separation was performed according to manufacturer instructions (StemCell technologies). Approximately 99% CD4^+^ or CD8^+^ cells were depleted from the corresponding negative fractions, as assessed by flow-cytometry using a FACS Calibur.

### IFN-γ ELISPOT

Millipore 96-wells flat-bottom plates (MAHA S4510, Polylabo) were incubated overnight at 4°C with 100 µL/well of capture anti-IFN-γ (MAb clone R4-6A2, BD-biosciences) at 10 µg/mL in PBS. Plates were washed 3 times with sterile PBS and incubated for 2 hr at 37°C with RPMI 10% FCS to prevent non specific binding. N antigen (100 µL/well of N SRS at 10 µg/mL in RPMI) was added to all wells and then 100 µl of serial doubling dilutions (starting from 2.5×10^6^ cells/mL) of freshly isolated total, CD4^−^ or CD8^−^ splenocytes in RPMI 10% FCS, PS, 2 mM L-glutamine and incubated 20 hr at 37°C in 5% CO_2_. The plates were washed with ultrapure water and then with PBS and incubated for 2 hr at 37°C with 100 µl/well of biotinylated anti-IFN-γ Mab (clone XMG1.2, BD biosciences) at 2 µg/mL in PBS supplemented with 0.05% Tween 20 and 1% BSA. After further washes and 45 min incubation with ExtrAvidin-alkaline-phosphatase at 1 µg/mL (Sigma), the IFN-γ producing cells were visualized by adding the substrate BCIP-NBT (Five-bromo-4-chloro-3-indolylphosphate/nitroblue tetrazolium; Sigma) in 100 mM Tris, 100 mM NaCl, 5 mM MgCl_2_, pH = 9.5 for 30 min. Results were expressed as the number of spot-forming cells (SFC) per 10^6^ input cells.

### N SRS-specific antibody E.L.I.S.A

Individual mouse sera and BAL supernatants were assayed for N-specific antibodies (total Ig, IgG2a, IgG1 and IgA) by ELISA. Microtiter plates (Immulon 2HB, ThermoLabsystems) were coated overnight at 4°C with N SRS antigen (200 ng per well in 100 µL carbonate-bicarbonate buffer 0.1 M pH 9.5). Plates were washed five times with PBS 0.05% Tween 20 between each step of the assay. After coating, the remaining protein binding sites were saturated with 5% FCS in PBS 0.05% Tween 20 (PBS-T-FCS) for 1 hr at 37°C. Samples were serially diluted threefold in PBS-T-FCS starting at 1:30 for sera and 1:3 for BAL and incubated for 2 hrs at 37°C. Antigen-bound Abs were detected using HRP-conjugated goat anti mouse IgH+L (P.A.R.I.S.) or rat anti mouse IgG1 or IgG2a (BD-Biosciences) or rat anti mouse IgA (Caltag) all at 1ng/mL, incubated for 1 hr at 37°C. The TMB substrate (Kirkegaard & Perry Laboratories Inc.) was added and the reaction was stopped after 10 min by 1M phosphoric acid. The absorbance was measured at 450 nm with an ELISA plate reader (Dynex, MRX revelation). The results were expressed as end-point antibody titers calculated by regression analysis plotting dilution versus A_450_ (regression curve y = (b+cx)/(1+ax) using Origin software). Endpoint titers were calculated as the highest dilution giving twice the absorbance of negative control.

Alternatively, plates were coated with heat-inactivated frozen-thawed cellular pellets from RSV-infected or mock-infected HEp-2 cultures in PBS. Samples were serially diluted and incubated with RSV-infected and control HEp-2 lysates, and the ELISA was performed as described above. To measure anti-RSV specific binding, background antibody binding to control HEp-2 lysate was deduced from binding to RSV-infected HEp-2.

### Data analysis

All data were expressed as arithmetic mean±standard error of the mean (SEM). Statistical analysis was performed using non parametric Mann-Whitney U test (http://elegans.swmed.edu/leon/stats/utest.html). Levels of significance are indicated on the graphs with stars: * p<0.05, ** p<0.01.
